# Very Long Chain Polyunsaturated Fatty Acids Accumulated in Triacylglycerol Are Channeled From Phosphatidylcholine in *Thraustochytrium*

**DOI:** 10.3389/fmicb.2019.00645

**Published:** 2019-03-27

**Authors:** Xianming Zhao, Xiao Qiu

**Affiliations:** Food and Bioproduct Sciences, University of Saskatchewan, Saskatoon, SK, Canada

**Keywords:** *Thraustochytrium*, lipid metabolism, very long chain polyunsaturated fatty acids, triacylglycerol biosynthesis, phosphatidylcholine biosynthesis, fatty acid trafficking

## Abstract

*Thraustochytrium* is a marine protist that can accumulate a large amount of very long chain polyunsaturated fatty acids (VLCPUFA) in triacylglycerols (TAG). How these freshly synthesized VLCPUFAs are channeled into TAG remains unknown. In this study, the glycerolipid profile of *Thraustochytrium* at log and stationary growth stages was first analyzed by lipidomic tools, and then ^14^C-acetate and ^14^C-glycerol were used to trace the flux of fatty acids and backbone in glycerolipids. Lipidomic analysis showed that VLCPUFAs were mostly allocated to phosphatidylcholine (PC) and TAG. PC possessed a relatively stable profile of VLCPUFAs, whereas TAG carrying VLCPUFAs were significantly increased at the stationary phase. ^14^C-acetate labeled VLCPUFAs were predominately incorporated into PC initially but were mostly found in TAG at later time of labeling. Positional analysis showed that PC had either one VLCPUFA at its *sn*-2 position (PC1) or two VLCPUFAs (PC2), while TAG incorporated VLCPUFAs almost exclusively at the *sn*-2 position. Similarly, ^14^C-glycerol was more efficiently incorporated into PC1 than TAG initially but was mostly found in TAG at later time of labeling, and diacylglycerol and PC1 shared a similar incorporation pattern. These results indicate that VLCPUFAs in TAG are mainly channeled from PC likely through diacylglycerol as the intermediate.

## Introduction

Omega-3 very long chain polyunsaturated fatty acids (VLCPUFA) such as docosahexaenoic acid (DHA) and eicosapentaenoic acid (EPA) have drawn increasing attention due to their potential roles in health promotion and disease prevention (Shahidi and Ambigaipalan, 2018). Currently, the major sources of such fatty acids are marine alga and fish oil. However, the *de novo* biosynthesis of VLCPUFAs occurs mainly in microorganisms such as algae, bacteria and fungi through two metabolic pathways (Qiu, 2003). The aerobic pathway goes through alternating elongations and desaturations to introduce double bonds and to extend the chain length of long chain fatty acids, while the anaerobic pathway utilizes a single multifunctional enzyme called PUFA synthase to synthesize VLCPUFAs from the initial precursor acetic acid. After synthesis, VLCPUFAs are mainly destined to two types of glycerolipids, phospholipids (PL) and triacylglycerols (TAG). Phospholipids such as phosphatidylcholine (PC), phosphatidylethanolamine (PE), phosphatidylinositol (PI), phosphatidylglycerol (PG), and phosphatidylserine (PS) are membrane lipids functioning to maintain the integrity and dynamics of cellular membrane systems, while TAG is the main storage form of glycerolipids serving as carbon and energy reserves.

The biosynthetic pathways of PL and TAG have been elucidated using isotope tracers, a powerful technique probing the relationships of initial substrates and final products (Kennedy, 1961, 1989; Kresge et al., 2005). For the biosynthesis of a group of phospholipids such as PS, PG and PI, phosphatidic acid (PA) serves as the backbone precursor for the attachment of a head group through an intermediate cytidine diphosphate-diacylglycerol (CDP-DAG). For the biosynthesis of another group of phospholipids such as PC and PE, DAG serves as the backbone precursor for the attachment of a head group donated by CDP-choline or CDP-ethanolamine. For the biosynthesis of TAG, two fatty acids are sequentially acylated to glycerol-3-phosphate at *sn*-1 and *sn*-2 positions, giving PA, which is then converted to DAG by removing the phosphate group at the *sn*-3 position. DAG is finally acylated with a third fatty acid at the *sn*-3 position to form TAG. As DAG is a central intermediate for both PC and TAG, efforts have recently been made to elucidate their relationship in plants (Bates et al., 2009; Bates and Browse, 2012). However, the exact relationship among DAG, PC and TAG is still not well understood, particularly for those marine microorganisms accumulating nutritionally important VLCPUFAs in TAG.

*Thraustochytrium* is a marine protist that can produce a relatively large amount of VLCPUFAs, mostly DHA. Therefore, *Thraustochytrium* and other related species have attracted much scientific and industrial interest in the production of DHA for commercial uses through fermentation and metabolic engineering (Patil and Gogate, 2015; Chandrasekaran et al., 2018; Chen and Yang, 2018; Sun et al., 2018). Our previous work indicates that DHA is solely synthesized by the anaerobic pathway using a polyketide synthase-like PUFA synthase comprising three large subunits each with multiple functional domains, whereas the aerobic pathway employing desaturases and elongases is incomplete in *Thraustochytrium* (Meesapyodsuk and Qiu, 2016; Zhao and Qiu, 2018). This study investigated how freshly synthesized VLCPUFAs were channeled into TAG for storage using pulse-chase and steady-state labeling of fatty acids and the glycerol backbone in *Thraustochytrium*. The results reveal that VLCPUFAs are mainly channeled to TAG from PC through DAG as the intermediate in *Thraustochytrium*. The finding of this unusual route of channeling VLCPUFAs to storage lipids not only contributes to the understanding of the acyl assembly during the biosynthesis of glycerolipids in these types of microorganisms, but also helps design strategies to improve the flux process for the heterologous production of these nutritionally important fatty acids in other eukaryotes.

## Materials and Methods

### Cultivation of *Thraustochytrium*

*Thraustochytrium* sp. 26185 was purchased from the American Type Culture Collection. It was maintained on the BY^+^ agar plate containing 0.1% yeast extract (w/v), 0.1% peptone (w/v), and 0.5% D-glucose in assimilated sea water. It was cultured with shaking in the GY medium consisting of 1% (w/v) yeast extract, 3% (w/v) D-glucose, and 1.75% (w/v) artificial sea salts (Sigma) at 25°C.

### Lipidomic Analysis

The total lipids were extracted following the previously reported method (Zhao and Qiu, 2018), and analyzed by Kansas Lipidomics Research Center Analytical Laboratory (KLRC, KS, United States) using electrospray ionization (ESI) triple quadrupole mass spectrometry (API 4000, Applied Biosystems, CA, United States) at the Kansas Technology Enterprise Corporation Analytical Laboratory, Kansas State University (Lee et al., 2011).

### Acyl Flux Analysis With Radiolabeled Acetic Acid and Glycerol Tracers

A single colony of *Thraustochytrium* was inoculated into 5 mL of GY medium and incubated at ambient temperature for 24 h before it was sub-cultured into 100 mL of fresh GY medium. When the culture reached early log phase (OD_600_ about 0.5), the cells were harvested by centrifugation at 4,000 rpm at 4°C for 5 min and re-suspended in fresh GY medium to an OD_600_ of 0.5. The culture was then incubated at ambient temperature for 1 h for equilibration before adding tracers for pulse-chase labeling or steady-state labeling.

For pulse-chase labeling by acetate, 10 μCi of [1-^14^C]-sodium acetate (50.5 μCi/μmol, PerkinElmer) was added to a 10-mL culture. After labeling for 30 min, the *Thraustochytrium* cells were immediately precipitated by centrifugation and washed twice with 10 mL of distilled water. Afterward, the cultivation was resumed in 10 mL of GY medium for 3 days. At 30 min of feeding and 3-days post feeding, the total lipids were extracted from the culture samples and used for lipid class, fatty acid composition and positional analysis. For pulse-chase labeling by glycerol, the same procedure was followed except that 100 μCi of [1,3-^14^C]-glycerol (55 μCi/μmol, American Radiolabeled Chemicals, Inc.) was used and the labeling time was 1 h instead of 30 min. No positional analysis was performed for ^14^C-glycerol labeled lipids.

For steady-state labeling by acetate, 10 μCi of [1-^14^C]-sodium acetate was added to a 20-mL culture. At the time point of 1, 2, 5, 10, 30, and 60 min, 6, 12, and 24 h after feeding, 1 mL of culture was harvested and used for lipid analysis. For steady-state labeling by glycerol, 50 μCi of [1,3-^14^C]-glycerol was fed to a 10-mL culture. At the time point of 10, 30, and 60 min, 6, 12, and 24 h of feeding, 1 mL of culture was harvested and used for lipid analysis. Three biological replicates were performed for all labeling experiments.

### Lipid Analysis

All methods used for lipid analysis were described in detail previously (Zhao and Qiu, 2018) unless stated otherwise. In brief, total lipids were extracted by a modified Bligh and Dyer method (Bligh and Dyer, 1959; Zhao and Qiu, 2018). Total lipids were separated into TAG, phospholipids (PL) and DAG on thin layer chromatography (TLC) plates by hexane/diethyl ether/acetic acid (70/30/1, v/v/v). Separated lipids were scraped from TLC plates and extracted from the silica with chloroform/methanol/water (5/5/1, v/v/v) and then followed by phase separation and collection of chloroform. PC was resolved from eluted PL on TLC plates with chloroform/methanol/acetic acid/water (75/25/4/4, v/v/v/v). For fatty acid analysis, fatty acid methyl esters (FAMEs) were prepared from scraped silica gels containing each lipid class by heating at 85°C for 1 h with 2% sulfuric acid in methanol. FAMEs were then separated into VLCPUFAs and SFAs on TLC plates pretreated with 10% AgNO_3_ in acetonitrile and developed with hexane/diethyl ether/acetic acid (94/4/2, v/v/v).

Positional analysis of TAG was conducted by a partial digestion using lipase from *Rhizomucor miehei* (Cahoon et al., 2006), which de-acylates TAG at the *sn*-1 and *sn*-3 position, producing free fatty acids (FFAs) and monoacylglycerol (MAG). The digestion products were then transmethylated by heating at 85°C for 1 h with 2% sulfuric acid in methanol and the resulted FAMEs were then separated by AgNO_3_-TLC. TAG was dissolved in 1 mL of diethyl ether, and 0.8 mL of a buffer containing 50 mM of borate and 5 mM of CaCl_2_ (pH 7.8) and 200 μL of lipase from *R. miehei* (Sigma L4277) were then added for digestion. The sample was mixed thoroughly by vortexing and incubated at 37°C for 1 h with shaking at 250 rpm. The reaction was stopped by adding 2 mL of methanol/chloroform (1/1, v/v). The mixture was centrifuged at 2,200 rpm for 10 min and the chloroform phase containing lipids was removed. The mixture was then back extracted with 2 mL of chloroform. The combined chloroform was dried under N_2_ gas and the digested lipids were resolved on TLC plates developed with hexane/diethyl ether/acetic acid (70/30/1, v/v/v). The fatty acid composition of resulted FFAs and MAG was analyzed by AgNO_3_-TLC as described above.

Positional analysis of PC was accomplished via the digestion of PC by phospholipase A2 (PLA2) following a previously reported method (Bates et al., 2007) with slight modifications. PLA2 was used to remove fatty acids at the *sn*-2 position of PC, producing FFAs and 1-acyl-lysophosphatidylcholine (LPC). PC was dissolved in 1 mL of diethyl ether, and then 0.1 mL of 50 mM Tris–HCl containing 5 mM of CaCl_2_ (pH 8.7), and 1 μL of PLA2 from porcine pancreas (Sigma P6534) were added for digestion. The sample was mixed vigorously at room temperature for 30 min. Afterward the ether was evaporated under N_2_ gas, and 3.8 mL of chloroform/methanol (2/1, v/v) and 1 mL of 0.15 M acetic acid were added. The chloroform phase was collected after centrifugation at 2,200 rpm for 10 min, and then 2.5 mL of chloroform was used to back extract the mixture. The combined chloroform was dried under N_2_ gas and the digested lipids were resolved into PC, LPC and FFAs on TLC plates developed with chloroform/methanol/acetic acid/water (50/30/8/4, v/v/v/v). The fatty acid composition of the resulting FFAs and LPC were analyzed by AgNO_3_-TLC as described above.

### Radioactivity Measurement

Radioactive bands on TLC plates were revealed by a phosphor-imaging method. The TLC plate as developed above was exposed to a storage phosphor screen (Amersham Biosciences) for at least 4 h. The screen was then visualized by a Typhoon FLA 7000 scanner (GE Health Care Life Sciences) equipped with an IP filter. Radioactivity of lipids by ^14^C-acetate labeling was determined by scintillation counting of FAMEs, which were eluted from AgNO_3_-TLC with hexane/isopropanol/water (60/40/5, v/v/v) following a previously reported method (Zhao and Qiu, 2018). Radioactivity of glycerolipids from ^14^C-glycerol labeling was determined by counting the radioactivity in the aqueous phase after transmethylation and extraction with hexane. Radioactivity as disintegration per minute (DPM) was measured by a liquid scintillation analyzer (Tri-carb 2910 TR, Perkin Elmer, Waltham, MA, United States).

## Results

### Lipidomic Analysis of *Thraustochytrium* Glycerolipids

**FIGURE 1:**
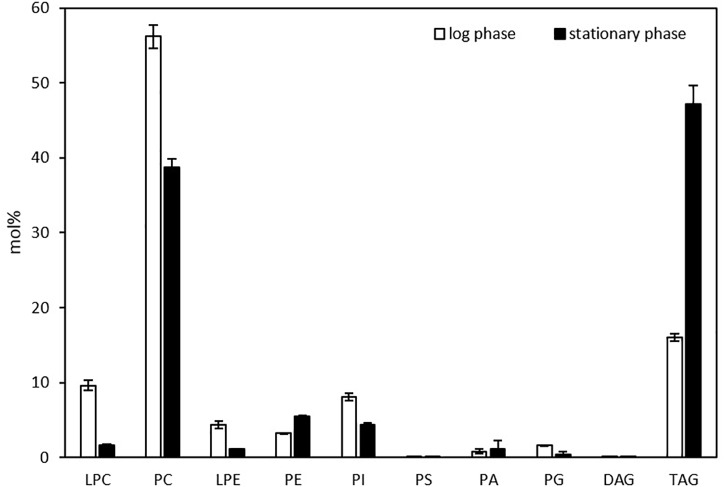
Glycerolipid composition in *Thraustochytrium* at the log and stationary growth phases. The values are means ± SD from three biological replicates.

Liquid chromatography-mass spectrometry was exploited to profile glycerolipids in one-day and three-day cultures of *Thraustochytrium,* corresponding to the log and stationary phases of growth (Meesapyodsuk and Qiu, 2016). As shown in Figure 1, *Thraustochytrium* produced a higher amount of polar lipids such as PC than neutral lipids such as TAG and DAG in the log phase of growth. At this stage, PC was the most abundant (56.2%) among the glycerolipids while TAG accounted for only 16.0% of the total glycerolipids, followed by LPC (9.6%), and other PLs such as PI, PE, PG, and PA. As the culturing time increased, the relative amount of PC decreased and TAG increased concurrently. At the stationary phase, TAG increased to 47.2% of the total glycerolipids, whereas PC decreased to 38.7%. This result indicates *Thraustochytrium* is active in synthesizing and pooling two types of glycerolipids, PC and TAG, and the biosynthesis of membrane lipid PC is robust at the log phase, and TAG is largely produced at the stationary stage. Interestingly, the pool of lysophosphatidylcholine (LPC) was also found to be much higher in the log phase than that in stationary phase, implying that the active biosynthesis of PC at the early growth stage might go through the LPC acylation pathway in *Thraustochytrium* (Lands, 1958).

**FIGURE 2:**
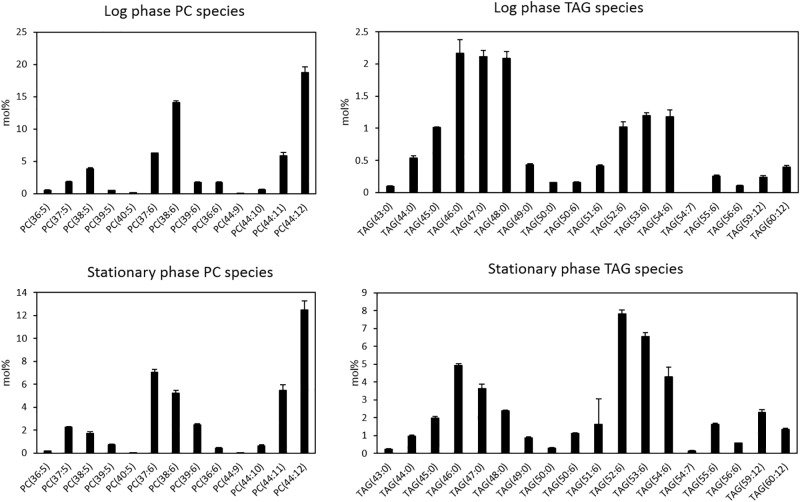
Distributions of PC and TAG species of *Thraustochytrium* at log and stationary growth phases. The data were presented as the molar percentage of each species in the total glycerolipids. The values are means ± SD from three biological replicates.

Next, we looked at distribution of molecular species of LPC, PC, and TAG in *Thraustochytrium* at the two growth phases. As shown in Figure 2, *Thraustochytrium* produced a unique fatty acid profile with two groups of fatty acids, VLCPUFAs (22:6n-3, 22:5n-6, and 20:5n-3) and long chain saturated fatty acids (SFAs) (15:0, 16:0, 17:0), which made it easy to determine PC and TAG species. DHA and DPA were the major fatty acids in LPC, together accounting for 97.7 and 89.8% of the total fatty acids at the log and stationary growth phases, respectively (data not shown). This result indicates that LPC significantly contribute to the accumulation of VLCPUFAs in PC. All PC species observed at the two stages contained at least one of three VLCPUFAs and no PC species with two SFAs were detected. In contrast, all TAGs at the two stages contained at least one SFA, and no TAG species with three VLCPUFAs were detected. Little difference was observed in the distribution of PC species between the two growth phases except for the increase of odd-chain SFA-containing PC species (37:6, 39:6, 37:5, and 39:5) with the concurrent decrease of even-chain SFA-containing PC species (38:6 and 38:5) at the stationary phase, which coincides well with our previous finding that *Thraustochytrium* could produce a large amount of odd-chain SFAs at a late growth stage (Zhao and Qiu, 2018). The abundant PC species observed at both stages were those comprising fatty acids equaling 44:12, indicating there were two DHA molecules acylated to these PC species. In contrast, much difference was observed in the distribution of TAG species between the two growth phases. The prevailing TAG species at the log phase were those with three SFAs, while TAG species with one DHA (52/53/54:6) dominated at the stationary phase. TAG species with three SFAs decreased from 63.4 to 35.8%, while TAG species with one VLCPUFA increased from 31.9 to 55.4% and TAG species with two VLCPUFAs increased from 4.7 to 8.8% over the two growth phases. Such shift of TAG species directly reflects the increased flux of VLCPUFAs into TAG at the late stage and an active acyl mobilization between PC and TAG might occur during the two growth stages.

### Acyl Flux Among Glycerolipids Traced by ^14^C-Acetate

To examine acyl flux among glycerolipids particularly between PC and TAG, radiolabeled ^14^C-acetate was exploited to trace the incorporation of freshly synthesized fatty acids into these glycerolipids using two different protocols, pulse-chase labeling and steady-state labeling. In pulse-chase labeling, *Thraustochytrium* was fed with ^14^C-acetate for 30 min. After that, unconsumed radiolabeled precursors were washed away, leaving the cells to grow for another three days. At 30 min of labeling and 3 days post labeling, the total glycerolipids in *Thraustochytrium* were analyzed. This protocol was used to examine the ultimate precursor-product relationships of the glycerolipids. In steady-state labeling, *Thraustochytrium* was fed with ^14^C-acetate continuously for 24 h. The total glycerolipids in *Thraustochytrium* were analyzed at nine time points (1 min, 2 min, 5 min, 10 min, 30 min, 60 min, 6 h, 12 h, and 24 h) over the time course. This protocol was used to trace the initial incorporation of freshly synthesized fatty acids and acyl trafficking in the glycerolipids.

#### Pulse-Chase Labeling by ^14^C-Acetate

**FIGURE 3:**
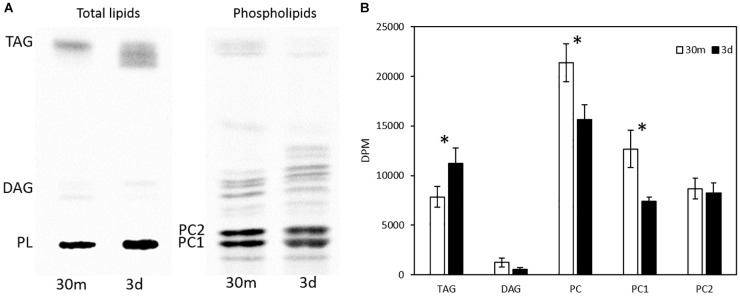
Radioactivity distribution in different glycerolipids in pulse-chase labeling by ^14^C-acetate. **(A)** TLC separation of total lipids into TAG, DAG, PC1, and PC2; **(B)** Radioactivity in TAG, DAG, PC, PC1, and PC2. The values are means ± SD from three biological replicates. ^∗^ indicates significant difference (*P* < 0.05).

The total glycerolipids in *Thraustochytrium* at two time points (30 min and 3 days post labeling) were extracted and separated into TAG, DAG, and PL firstly on a TLC plate, and the PL was then extracted from the plate and further separated on another TLC plate into different phospholipid subclasses, mainly two types of PC, PC1 (PC with one VLCPUFA and one SFA) and PC2 (PC with two VLCPUFAs) (confirmed by positional analysis, see below). As shown in Figure 3, the total radioactivity of labeled fatty acids in major glycerolipids including TAG, DAG, PC1, and PC2 remained stable over the two time points. At both time points, most of the labeled radioactivity was found in TAG and PC, together accounting for more than 95% of the total radioactivity, whereas DAG incorporated less than 5% of the total radioactivity. Between the two time points, significant changes in the amount of radioactivity were observed in PC and TAG. At three days post labeling, radioactivity in TAG increased significantly (*P* < 0.05) while that in PC decreased significantly (*P* < 0.05). Such concurrent changes in the radioactivity of PC and TAG are clearly indicative of an active acyl trafficking between PC and TAG between the two time points. Furthermore, the decrease of radioactivity in PC at three days post labeling was mainly observed on PC1, not on PC2 where the radioactivity at the two time points remained relatively stable, implying that the acyl trafficking might mainly take place between PC1 and TAG between the two time points.

**FIGURE 4:**
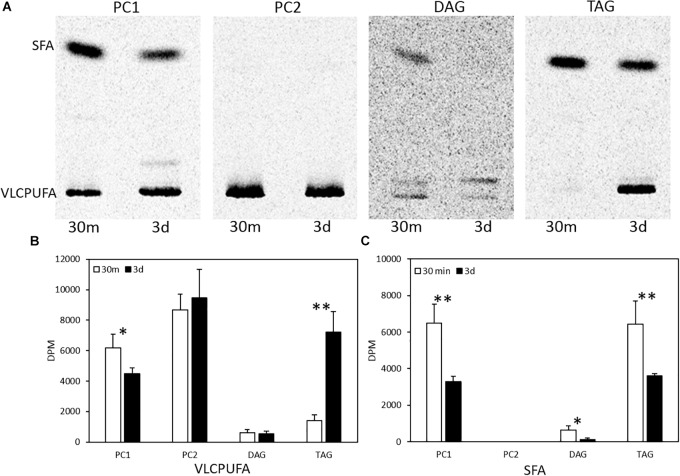
Quantification of saturated and polyunsaturated fatty acids in glycerolipids in pulse-chase labeling by ^14^C-acetate. **(A)** AgNO_3_-TLC separation of labeled fatty acids in PC1, PC2, DAG, and TAG; **(B)** Radioactivity of VLCPUFAs in the glycerolipids; **(C)** Radioactivity of SFAs in the glycerolipids. The values are means ± SD from three biological replicates. ^∗^ indicates significant difference (*P* < 0.05) and ^∗∗^ indicates significant difference (*P* < 0.01).

To examine fatty acid profiles of glycerolipids, each lipid class was scraped from TLC plates and transmethylated to FAMEs, which were then separated into two groups of fatty acids, VLCPUFAs and SFAs, by AgNO_3_-TLC. As shown in Figure 4, a very different pattern of ^14^C labeled fatty acid accumulation in PC, DAG and TAG was observed. PC1 accumulated more ^14^C labeled SFAs and VLCPUFAs at 30 min of labeling than at three days post labeling. PC2 accumulated exclusively ^14^C labeled VLCPUFAs and remained relatively stable at both time points. DAG accumulated some ^14^C labeled SFAs at 30 min of labeling, but very little of them at three days post labeling. On the other hand, TAG accumulated almost exclusively ^14^C labeled SFAs at 30 min of labeling, whereas at three days post labeling it accumulated more ^14^C labeled VLCPUFAs than SFAs. The amounts of ^14^C labeled VLCPUFAs in PC2 and DAG remained relatively constant, while those in PC1 and TAG were significantly different between the two time points. At 3-days post labeling, the amount of ^14^C labeled VLCPUFAs in PC1 decreased by 27.7%, while the amount of ^14^C labeled VLCPUFAs in TAG increased by about four times. This result clearly indicates the occurrence of VLCPUFA flux from PC1 to TAG.

**FIGURE 5:**
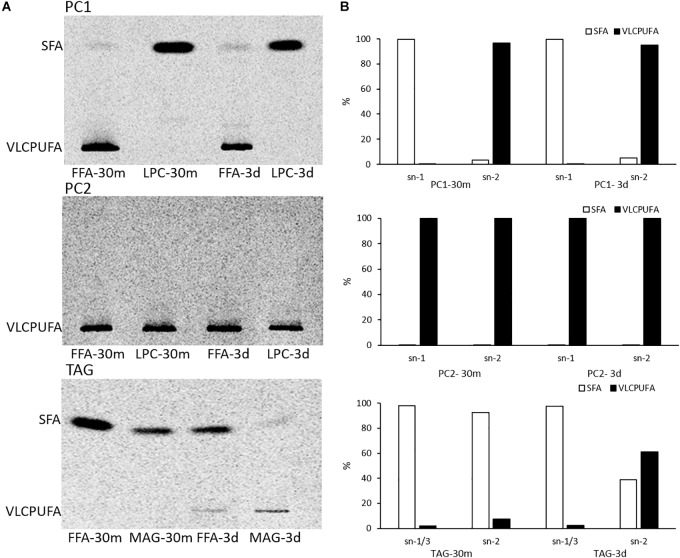
Positional analysis of PC1, PC2, and TAG in pulse-chase labeling by ^14^C-acetate. **(A)** AgNO_3_-TLC separation of FAMEs of digested PC1, PC2, and TAG products; **(B)** quantitative analysis of fatty acid distribution in PC1, PC2, and TAG.

To investigate possible stereospecific positions of glycerolipids involved in acyl trafficking, positional analysis of PC1, PC2, and TAG from pulse-chase labeling was performed. TAG was partially digested by lipase from *R. miehei*, generating two FFAs from *sn*-1 and *sn*-3 positions and one 2-acyl-*sn*-glycerol (monoacylglycerol, MAG). PC1 and PC2 were digested by a phospholipase A2 from porcine pancreas, generating one FFA from *sn*-2 position and one 1-acyl-*sn*-glycerol-3-phosphocholine (α-lysophosphatidylcholine, LPC). Afterward, the fatty acid profiles of digestion products were resolved by AgNO_3_-TLC. As shown in Figure 5, PC1 at both time points comprised one VLCPUFA exclusively located at the *sn*-2 position and one SFA exclusively located at the *sn*-1 position. PC2 at both time points comprised two VLCPUFAs with one at the *sn*-1 position and the other at the *sn*-2 position. At 30 min of labeling, ^14^C labeled TAG comprised almost exclusively ^14^C labeled SFAs, and all the three positions were occupied by SFAs. However, at three days post labeling, TAG accumulated a substantial amount of ^14^C labeled VLCPUFAs that were almost exclusively located at the *sn*-2 position, while the SFAs were preferentially located at the *sn*-1/3 positions. This result indicates that the trafficking of VLCPUFAs between PC1 and TAG might mainly occur at the *sn*-2 position of the two.

#### Steady-State Labeling by ^14^C-Acetate

**FIGURE 6:**
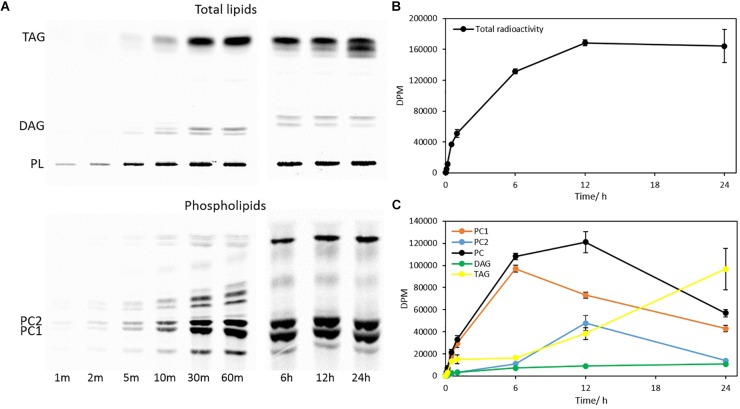
Incorporation of ^14^C-acetate label into fatty acids of different glycerolipids in a time course of 24 h. **(A)** TLC separation of total glycerolipids into TAG, DAG and PL, and PL into PC1 and PC2; **(B)** Total radioactivity in glycerolipids; **(C)** Radioactivity in PC1, PC2, total PC, DAG, and TAG. The values are means ± SD from three biological replicates.

To interrogate when and how initial acyl trafficking occurs among glycerolipids, a steady-state labeling experiment of *Thraustochytrium* was carried out over a period of 24 h using ^14^C-acetate. The total glycerolipids from the *Thraustochytrium* cultures at nine time points (1 min, 2 min, 5 min, 10 min, 30 min, 60 min, 6 h, 12 h, and 24 h) were extracted and separated into TAG, DAG, and PL. Eluted PL was then separated into PC1, PC2, and other PLs. As shown in Figure 6, the total incorporation of radioactivity in glycerolipids occurred at a fast linear rate over the first one hour and reached the maximum at 12 h of labeling. After that, the total radioactivity in glycerolipids remained relatively constant. Similar to the pulse-chase labeling, most of the labeled fatty acids went into two major glycerolipids PC and TAG with a small amount into DAG throughout the labeling course. The initial incorporation rate in the first minute of labeling was 2.10, 1.27, and 0.01 nmol of ^14^C-acetate for PC, TAG, and DAG, respectively, with PC being the fastest and DAG being the least in labeling. At 12 h of labeling, PC (PC1 + PC2) accumulated almost two thirds of the total radioactivity in glycerolipids. This result indicates that freshly synthesized fatty acids were more efficiently incorporated into PC than TAG initially, particularly in the first 6 h. At 12 h of labeling, radioactivity in PC reached the maximum; afterward the radioactivity started to decrease. On the other hand, incorporation of labeled fatty acids into TAG was slow over the initial period of 6 h. After that, the incorporation started to pick up and surpassed PC at 24 h of labeling. Such shift of the incorporated radioactivity in PC and TAG reaffirms that acyl trafficking occurs between PC and TAG as observed in the pulse-chase labeling. A further comparison of radioactivity in PC1 and PC2 showed that incorporation of freshly synthesized fatty acids was much faster in PC1 than PC2, particularly over the first 6 h of labeling. After that, radioactivity in PC1 started to decrease. However, radioactivity in PC2 increased continuously after 6 h and reached the maximum at 12 h. Nevertheless, radioactivity in PC1 remained higher than that in PC2 throughout the entire labeling course. Interestingly, while PC1 started to lose radioactivity after 6 h, TAG and PC2 started to pick up the radioactivity. Such a shift of radioactivity accumulation suggests that PC1 is the major donor of acyl groups to TAG and PC2 and the acyl trafficking starts rapidly only after the 6 h of labeling.

**FIGURE 7:**
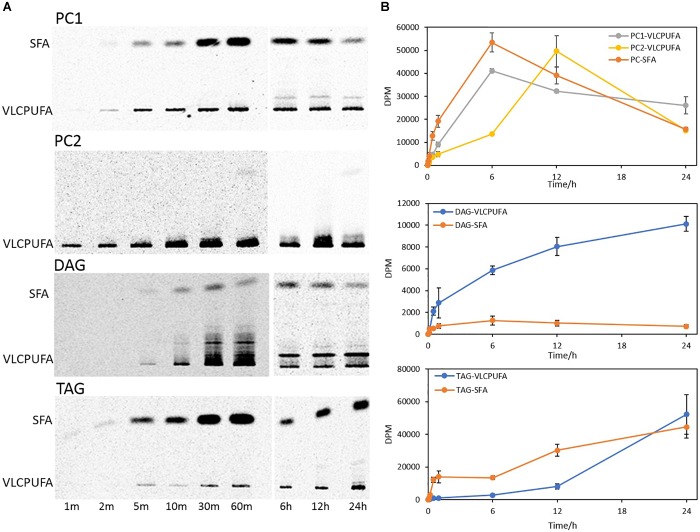
Incorporation of ^14^C-acetate label into VLCPUFAs and SFAs of different glycerolipid classes. **(A)** AgNO_3_-TLC separation of PC1, PC2, DAG, and TAG FAMEs; **(B)** Radioactivity of VLCPUFAs and SFAs in PC, DAG, and TAG. The values are means ± SD from three biological replicates.

To examine fatty acid profiles of glycerolipids, PC, TAG, and DAG were scraped from the TLC plate and transmethylated to FAMEs, which were then separated into VLCPUFAs and SFAs by AgNO_3_-TLC. As shown in Figure 7, DAG accumulated a higher amount of ^14^C labeled VLCPUFAs than ^14^C labeled SFAs throughout the time course, whereas TAG accumulated a higher amount of ^14^C labeled SFAs than ^14^C labeled VLCPUFAs over almost the entire time course except at 24 h of labeling. PC1 incorporated ^14^C labeled VLCPUFAs and ^14^C labeled SFAs at a similar rate in the first 6 h, and after that it started to lose both fatty acids. On the other hand, incorporation of ^14^C labeled VLCPUFAs in PC2 was slow in the first 6 h, and after that it started to grow rapidly, and reached the maximum at 12 h of labeling, after which it also started to lose VLCPUFAs dramatically.

**FIGURE 8:**
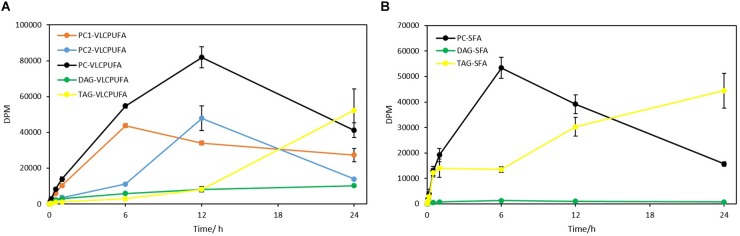
Quantitative analysis of ^14^C-acetate label into VLCPUFAs and SFAs of different glycerolipids. **(A)** radioactivity of ^14^C-VLCPUFAs in PC1, PC2, total PC, DAG, and TAG; **(B)** radioactivity of ^14^C-SFAs in PC, DAG, and TAG. The values are means ± SD from three biological replicates.

A further comparison of fatty acid profiles in different glycerolipids revealed very different patterns of ^14^C labeled VLCPUFA incorporation (Figure 8A). PC entered an almost linear incorporation of ^14^C labeled VLCPUFAs for the first 6 h, whereas TAG accumulated ^14^C labeled VLCPUFAs considerably slower at the same period and collected only 7.6% of that in PC at 60 min. DAG collected more ^14^C labeled VLCPUFAs than TAG at 60 min, even though the total amount of radioactivity in DAG was only 21.7% of that in TAG at the same time point. After 6 h, the incorporation of VLCPUFAs in TAG increased and continued, while at the same time the PC lost VLCPUFAs, until TAG had accumulated the majority of the VLCPUFAs by 24 h. These results also indicate that SFAs were channeled along with VLCPUFAs from PC1 to TAG (Figure 8B).

### Backbone Flux Among Glycerolipids Traced by ^14^C-Glycerol

The above ^14^C-acetate labeling revealed an acyl trafficking between PC and TAG; however, it remained to be determined what would happen to the glycerol backbone when the acyl groups were transferred. Therefore, similar protocols of pulse-chase labeling and steady-state labeling by ^14^C-glycerol were also performed to trace the incorporation of the backbone among glycerolipids. When *Thraustochytrium* was provided with ^14^C-glycerol, both the backbone and acyl groups in glycerolipids were labeled, as glycerol could serve as a precursor for the synthesis of acetic acid, the precursor for the biosynthesis of fatty acids. Therefore, to trace the flux, only the radiolabeled backbone of glycerolipids was measured.

#### Pulse-Chase Labeling by ^14^C-Glycerol

**FIGURE 9:**
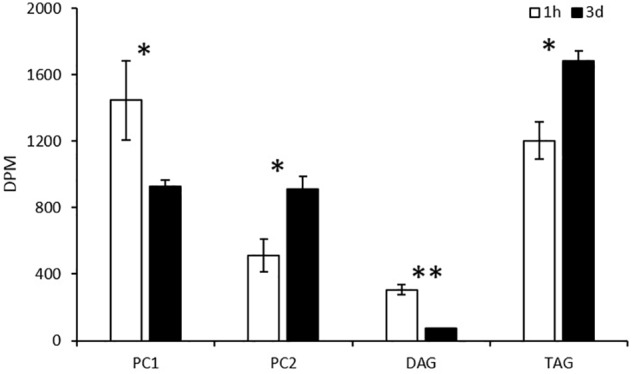
Incorporation of ^14^C-glycerol into PC1, PC2, DAG, and TAG as the backbone in pulse labeling. The values are means ± SD from three biological replicates. ^∗^ indicates significant difference of *P* < 0.05 and ^∗∗^ indicates significant difference of *P* < 0.01.

As shown in Figure 9, labeled glycerol predominately went into PC1, PC2, and TAG, whereas DAG accumulated only a small amount of radioactivity at both 1 h of labeling and 3-days post labeling. At 1 h of labeling, radiolabeled glycerol was more efficiently incorporated into PC1, and then TAG, PC2, and DAG. At 3-days post labeling, PC1 and DAG reduced backbone radioactivity while TAG and PC2 gained the radioactivity simultaneously. This result coincides with that of the acyl trafficking, revealing that the backbone is channeled along with fatty acids from PC1 to TAG.

#### Steady-State Labeling by ^14^C-Glycerol

**FIGURE 10:**
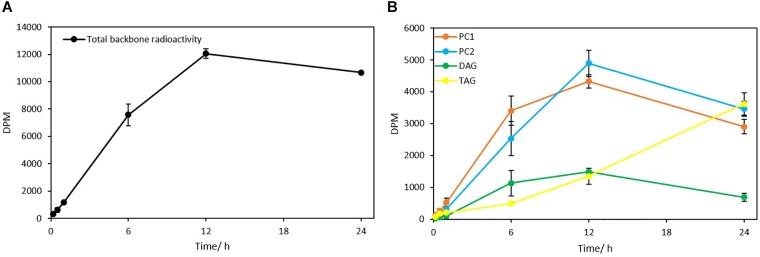
The incorporation of ^14^C-glycerol into different glycerolipid classes as the backbone. **(A)** Total radioactivity in glycerolipids as backbone; **(B)** Backbone radioactivity in PC1, PC2, DAG, and TAG. The values are means ± SD from three biological replicates.

The steady-state backbone labeling of *Thraustochytrium* was carried out over a period of 24 h. Total glycerolipids in *Thraustochytrium* at six labeling points (10 min, 30 min, 60 min, 6 h, 12 h, and 24 h) in a time course were extracted and separated into TAG, DAG, and PL, and eluted PL was further separated into PC1 and PC2. As shown in Figure 10, the total backbone incorporation into glycerolipids increased rapidly in the first 6 h and reached the peak at 12 h. The labeled glycerol went predominately into PC (PC1 + PC2), and the incorporation in DAG and TAG together accounted only for less than 20% of the total radioactivity at 12 h. Within PC, the incorporation in PC1 was more efficiently than that in PC2 initially. At 6 h of labeling, the radioactivity in PC1 was about 1.4 times of that in PC2. After that, the incorporation in PC1 slowed down, while the incorporation in PC2 speeded up. At 12 h, PC2 incorporated more radioactivity than PC1. On the other hand, TAG collected only 6.5% of the total radioactivity at 6 h, but after that, the incorporation speeded up and overtook all the other glycerolipids at 24 h. Interestingly, DAG displayed a similar incorporation pattern with PC1, despite it incorporated a much less amount of the backbone than PC1. At 6 h DAG accumulated about two times the radioactivity as seen in TAG. After 12 h, it started to lose the radioactivity, and by 24 h the radioactivity in DAG was only about 20% of that in TAG. These results suggest that the backbone trafficking mainly occurs between PC1 and TAG, such trafficking also takes place more vigorously after 6 h of labeling.

## Discussion

In the present study, lipidomic tools and two types of radioactive tracers were used to examine the trafficking process of VLCPUFAs among glycerolipids in *Thraustochytrium*. Lipidomic analysis of the glycerolipids in *Thraustochytrium* at log and stationary growth stages showed that VLCPUFAs were mostly pooled into two types of glycerolipids PC and TAG, and an increased mobilization of VLCPUFAs from PC to TAG may have occurred at the latter growth stage. To examine the possible acyl trafficking, ^14^C-acetate was first exploited to trace the flux of freshly synthesized fatty acids among glycerolipids. When *Thraustochytrium* was provided with a pulse of ^14^C-acetate for 30 min, freshly synthesized VLCPUFAs were initially incorporated into PC rather than TAG, but at three days post labeling, the incorporation in PC was decreased with a concurrent increase of the incorporation in TAG. This result confirms the occurrence of the VLCPUFA trafficking from PC to TAG. TLC analysis of ^14^C-acetate-labeled products showed PC in *Thraustochytrium* was comprised of two types, PC1 (with one SFA at *sn*-1 position and one VLCPUFA at *sn*-2 position) and PC2 (with VLCPUFAs at both *sn*-1 and *sn*-2 positions). It was clear that the decrease of the acyl radioactivity in PC at three days post labeling was mainly observed on PC1, not on PC2, indicating that the acyl trafficking mainly occurs from PC1 to TAG. Furthermore, the decrease of radioactivity in PC1 and the increase of radioactivity in TAG were mainly reflected on VLCPUFAs at the *sn*-2 position of the two glycerolipids, indicating that the VLCPUFA trafficking takes place from the *sn*-2 position of PC1 to the *sn*-2 position of TAG. When *Thraustochytrium* was provided with a steady supply of ^14^C-acetate over 24 h, freshly synthesized VLCPUFAs were more efficiently incorporated into PC1 than TAG where only a very small proportion of total VLCPUFAs were accumulated in the first 6 h of labeling. PC1 started to lose VLCPUFAs while TAG began to gain these fatty acids after 6 h of labeling. This result further indicates that the mobilization of freshly synthesized VLCPUFAs from PC1 to TAG occurs vigorously only after 6 h of labeling.

To interrogate the possible involvement of the glycerol backbone in the acyl trafficking between PC and TAG, ^14^C-glycerol was exploited to trace the backbone flux among the glycerolipids. When *Thraustochytrium* was provided with a pulse of ^14^C-glycerol for 1 h, the backbone was more efficiently incorporated into PC1 than TAG, PC2, and DAG. Three days after pulse feeding, the backbone was lost from PC1 and mostly incorporated in TAG, indicating an increased backbone flux from PC1 to TAG at the latter stage. When *Thraustochytrium* was provided with a steady supply of ^14^C-glycerol for 24 h, PC1 incorporated labeled glycerol much more efficiently than TAG during the first 6 h. After that, the incorporation slowed down. After 12 h, PC1 started to lose the radioactivity. On the other hand, TAG incorporated backbone less efficiently in the first 6 h, but after that, incorporation increased. At 24 h of labeling, the incorporation of the labeled backbone in TAG surpassed PC1. This result indicates that the backbone transfer between PC1 and TAG, coincided with acyl trafficking, occurs actively after 6 h of labeling.

Several lines of evidence point to the notation that DAG might be an intermediate transferred from PC1 to TAG. Firstly, the pulse-chase labeling of ^14^C-acetate and ^14^C-glycerol showed both SFAs and VLCPUFAs in PC1 were simultaneously channeled to TAG, coincident with the transfer of backbone that could carry these fatty acids from PC1 to TAG. Secondly, the steady-state labeling of ^14^C-acetate and ^14^C-glycerol showed the acyl and backbone trafficking between PC1 and TAG possessed a similar mobilization trend, actively occurring after 6 h of labeling in the time course. Thirdly, most VLCPUFAs-containing TAG species have similar stereospecific structure as PC1, i.e., SFA at the *sn*-1 position and VLCPUFA at the *sn*-2 position. Fourthly, the steady-state labeling of ^14^C-glycerol showed that DAG and PC1 shared a similar incorporation pattern, and the decrease of backbone radioactivity in PC1 and DAG are concurrent with the increase of the radioactivity in TAG. Collectively, these results indicate PC-derived DAG is likely an intermediate for the biosynthesis of TAG in *Thraustochytrium*.

There are two distinct pathways for the biosynthesis of TAG using DAG as a substrate in nature. In the acyl-CoA independent pathway, the synthesis of TAG is catalyzed by phospholipid:diacylglycerol acyltransferase (PDAT) transferring an acyl group from the *sn*-2 position of a phospholipid to the *sn*-3 position of DAG (Dahlqvist et al., 2000). In *Thraustochytrium*, a candidate gene encoding this enzyme was identified in the genome (Zhao et al., 2016). However, the fact that no VLCPUFA was found in TAG at 30 min of labeling, and only a very small amount of VLCPUFAs were found at the *sn*-1/3 positions of TAG at three days post labeling implies that PDAT might not play a major role in channeling VLCPUFAs to TAG in this protist. In acyl-CoA dependent pathway, biosynthesis of TAG is catalyzed by acyl-CoA:diacylglycerol acyltransferase (DGAT) transferring an acyl group from acyl-CoA to the *sn*-3 position of DAG. Our labeling results indicate that VLCPUFAs channeled to TAG do not come from acyl-CoA pool, but rather from the DAG backbone of PC1 in *Thraustochytrium*. In eukaryotes, DAG is generally believed to be derived from two sequential acylations at *sn*-1 and 2 positions catalyzed by glycerol-3-phosphate acyltransferase (GPAT) and lysophosphatidic acid acyltransferase (LPAT), followed by removing phosphate group at the *sn*-3 position by phosphatidic acid phosphatase (PAP) (Sorger and Daum, 2003). DAG derived from PC has rarely been reported in living organisms except for a few plant species where the biosynthesis of highly unsaturated and unusual fatty acids occurs on PC and converting PC to DAG is probably catalyzed by phosphatidylcholine:diacylglycerol choline transferase (PDCT) (Bates and Browse, 2011, 2012; Bates et al., 2012). In *Thraustochytrium*, candidate gene encoding PDCT was not found in the genome (Zhao et al., 2016). Therefore, it remains to be determined how DAG is derived from PC in this protist.

In eukaryotes, PC can be synthesized by a few pathways (Carman and Henry, 1999). However, the biosynthesis of VLCPUFA-containing PC in *Thraustochytrium* might mainly go through a LPC acylation pathway catalyzed by acyl-CoA:lysophosphatidylcholine acyltransferase (LPCAT) (Chen et al., 2007). Firstly, no genes encoding PDCT catalyzing the exchange of the head group between existing PC and DAG giving new PC and DAG was found in the genome (Zhao et al., 2016). Secondly, a small amount of PE present in *Thraustochytrium* might not be able to support a large capacity of PC synthesis through PE methylation. On the other hand, a high level of LPC rich in VLCPUFAs, the important intermediate of the LPC acylation pathway, was found in *Thraustochytrium* (Figure 2). Thirdly, the ^14^C-acetate-steady labeling showed that VLCPUFAs were initially incorporated into PC rather than DAG (Figure 3, 6), a substrate for PC biosynthesis through a head group activation pathway catalyzed by cholinephosphotransferase (CPT). In *Thraustochytrium*, candidate genes encoding LPCAT and glycerol-3 phosphocholine acyltransferase (GPCAT) were identified, although their functions have not been confirmed (Zhao et al., 2016). These two enzymes catalyze two sequential acylations of glycerol-3-phosphocholine (G3PC) in the LPC acylation pathway to form PC. In plants, the LPC acylation pathway is commonly used for acyl editing to provide PC-derived DAG with highly unsaturated and unusual fatty acids for the biosynthesis of TAG, as important fatty acid modifications such as desaturation, hydroxylation, epoxidation, and acetylation occur only on PC. However, in *Thraustochytrium*, VLCPUFAs are directly synthesized by a PUFA synthase, and the acyl-desaturation process on PC is deemed unnecessary. Thus, it remains to determine why VLCPUFAs are first incorporated to PC, and then channeled to TAG in *Thraustochytrium*.

**FIGURE 11:**
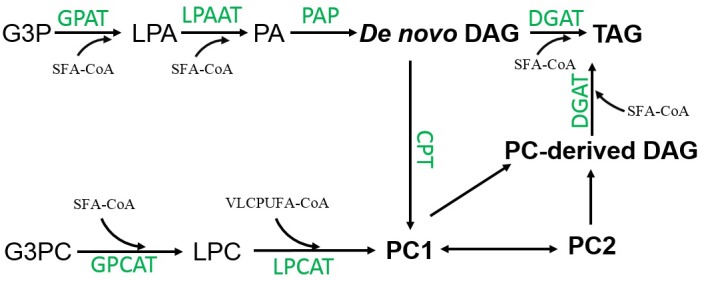
Possible pathways of acyl trafficking among glycerolipids in *Thraustochytrium*.

In summary, biosynthesis of SFA-TAG in *Thraustochytrium* at the early growth stage likely uses *de novo* DAG provided by the traditional pathway through two sequential acylations of glycerol-3-phosphate (G3P), while biosynthesis VLCPUFA-TAG at the late growth stage likely utilizes PC-derived DAG provided by an unknown mechanism. Freshly synthesized VLCPUFAs are initially incorporated into PC1 possibly through the GPC pathway where LPC plays a central role in accumulating VLCPUFAs, and DAG derived from this PC is a source of VLCPUFAs in TAG. In addition, PC1, to the less extent, can also be a precursor for PC2 probably through an acyl-editing process, and this PC species can also provide DAG for the biosynthesis of TAG (Figure 11). Future direction would be to elucidate how PC is converted to DAG, the key intermediate for the biosynthesis of VLCPUFAs-containing TAG in *Thraustochytrium*.

## Data Availability

All datasets generated for this study are included in the manuscript and/or the supplementary files.

## Author Contributions

XQ and XZ contributed to design of the study and writing of the manuscript. XZ also contributed to acquisition and analysis of the data.

## Conflict of Interest Statement

The authors declare that the research was conducted in the absence of any commercial or financial relationships that could be construed as a potential conflict of interest.
